# Biallelic inactivating variants in the chromatin remodeler *DMAP1* cause a syndromic neurodevelopmental disorder

**DOI:** 10.1172/JCI198229

**Published:** 2026-06-11

**Authors:** Qin Wang, Andrew K. Sobering, Christian Tirrito, Sadegheh Haghshenas, Tina Duelund Hjortshøj, Konrad Platzer, Silke Redler, Michael E. March, Leticia S. Matsuoka, Hang Xi, Josiah Zoodsma, Yuanhua Chen, Mari Mori, Marco L. Leung, Nathalie Couque, Alain Verloes, Antoine Pouzet, Noor A.A. Giesbertz, Marleen E.H. Simon, Ashley K. Yearwood, Dominique L. Assing, Tzung-Chien Hsieh, Jing-Mei Li, Michael A. Levy, Jennifer Kerkhof, Haley McConkey, Jessica Rzasa, Carolyn Lauzon-Young, Raashda A. Sulaiman, Firdous Abdulwahab, Hanan E. Shamseldin, Naif A.M. Almontashiri, Manal Afqi, Vettaikorumakankav Vedanarayanan, Maria J. Guillen Sacoto, Ingrid M. Wentzensen, Nadirah S. Damseh, Rivka Birnbaum, Babeth van Ommeren, Saskia M.J. Hopman, Maha S. Zaki, Gehad Elmakkawy, Erum Afzal, JiHye Kim, Stephanie Efthymiou, Henry Houlden, Ambreen Nusrat, Mathias Toft, Uzma Abdullah, Zafar Iqbal, Shannon Terek, Fowzan S. Alkuraya, Elizabeth J. Bhoj, Reza Maroofian, Bekim Sadikovic, Hakon Hakonarson, Yuanquan Song, Dong Li

**Affiliations:** 1 Raymond G. Perelman Center for Cellular and Molecular Therapeutics, Children’s Hospital of Philadelphia, Philadelphia, Pennsylvania, USA.; 2 Department of Basic Sciences, Augusta University/University of Georgia Medical Partnership, Athens, Georgia, USA.; 3 Windward Islands Research & Education Foundation, True Blue, St. George’s, Grenada.; 4 Department of Biochemistry, St. George’s University School of Medicine, St. George’s, Grenada.; 5 Biology Graduate Group, University of Pennsylvania, Philadelphia, Pennsylvania, USA.; 6 Verspeeten Clinical Genome Centre, London Health Sciences Centre, London, Ontario, Canada.; 7 Department of Clinical Genetics, University Hospital of Copenhagen, Copenhagen, Denmark.; 8 Institute of Human Genetics, University of Leipzig Medical Center, Leipzig, Germany.; 9 Institute of Human Genetics, Medical Faculty and University Hospital Düsseldorf, Heinrich-Heine-University Düsseldorf, Düsseldorf, Germany.; 10 Center for Applied Genomics, Children’s Hospital of Philadelphia, Philadelphia, Pennsylvania, USA.; 11 Emory University School of Medicine, Atlanta, Georgia, USA.; 12 The Steve and Cindy Rasmussen Institute for Genomic Medicine, Nationwide Children’s Hospital, Columbus, Ohio, USA.; 13 Departments of Pathology and Pediatrics, The Ohio State University College of Medicine, Columbus, Ohio, USA.; 14 Genetics Department, Robert Debré University Hospital, Assistance Publique–Hôpitaux de Paris, Paris, France.; 15 Multi-site Medical Biology Laboratory SeqOIA-FMG2025, Paris, France.; 16 French National Reference Centre for Developmental Anomalies and Malformative Syndromes of Île de France, Robert Debré University Hospital, Assistance Publique–Hôpitaux de Paris, Paris, France.; 17 Department of Genetics, Netherlands Cancer Institute, Amsterdam, Netherlands.; 18 Department of Genetics, University Medical Center Utrecht, Utrecht, Netherlands.; 19 Department of Radiology, UCLA, Los Angeles, California, USA.; 20 Institute for Genomic Statistics and Bioinformatics, University Hospital Bonn, Rheinische Friedrich-Wilhelms-Universität Bonn, Bonn, Germany.; 21 Department of Pathology and Laboratory Medicine, Western University, London, Ontario, Canada.; 22 Department of Medical Genomics, Center for Genomic Medicine, King Faisal Specialist Hospital and Research Center, Riyadh, Saudi Arabia.; 23 College of Medicine, Alfaisal University, Riyadh, Saudi Arabia.; 24 College of Applied Medical Sciences and Center for Genetics and Inherited Diseases, Taibah University, Madinah, Saudi Arabia.; 25 Research Department, King Khaled Eye Specialist Hospital, Riyadh, Saudi Arabia.; 26 Unit of Genetic Diseases, Department of Pediatrics, Maternity and Children’s Hospital, Almadinah Almunwarah, Saudi Arabia.; 27 The University of Texas at Austin Dell Medical School, Austin, Texas, USA.; 28 GeneDx Inc., Gaithersburg, Maryland, USA.; 29 Department of Pediatrics & Genetics, Makassed Hospital & Al-Quds Medical School, E. Jerusalem, Palestine.; 30 Department of Genetics, Hadassah Medical Center, Jerusalem, Israel.; 31 Clinical Genetics Department, Human Genetics and Genome Research Institute, National Research Centre, Cairo, Egypt.; 32 Human Genetics Department, Medical Research Institute, Alexandria University, Alexandria, Egypt.; 33 Department of Development Pediatrics, The Children’s Hospital and The Institute of Child Health, Multan, Pakistan.; 34 3billion Inc., Seoul, Korea.; 35 Department of Neuromuscular Disease, UCL Queen Square Institute of Neurology, London, United Kingdom.; 36 Aero Hospital, Wah Cantt, Pakistan.; 37 Department of Neurology, Oslo University Hospital, Oslo, Norway.; 38 Institute of Clinical Medicine, University of Oslo, Oslo, Norway.; 39 University Institute of Biochemistry and Biotechnology, Pir Mehr Ali Shah Arid Agriculture University, Rawalpindi, Pakistan.; 40 Ambry Genetics, Aliso Viejo, California, USA.; 41 Department of Anatomy and Cell Biology, College of Medicine, Alfaisal University, Riyadh, Saudi Arabia.; 42 Division of Human Genetics, Children’s Hospital of Philadelphia, Philadelphia, Pennsylvania, USA.; 43 Department of Pediatrics, University of Pennsylvania Perelman School of Medicine, Philadelphia, Pennsylvania, USA.; 44 Department of Genetics and; 45 Department of Pathology and Laboratory Medicine, University of Pennsylvania, Philadelphia, Pennsylvania, USA.

**Keywords:** Development, Genetics, Neuroscience, Epigenetics, Neurodevelopment

## Abstract

Chromatin remodeling is a dynamic epigenetic process that alters chromatin structure to gauge gene accessibility, enabling precise spatiotemporal gene expression, with disruptions often underlying neurodevelopmental disorders (NDDs), although the mechanistic underpinning remains incompletely understood. Despite essential roles in chromatin remodeling processes such as DNA methylation and histone acetylation and deposition, DMAP1 has not been implicated in human disease. We identified 20 individuals from 16 families with a syndromic NDD carrying homozygous or compound heterozygous variants in *DMAP1*. Neural-specific knockdown of its *Drosophila* ortholog, *dDMAP1*, caused pupal lethality, structural defects in the mushroom body (MB), decreased dendrite length, abnormal social behavior and mechanical-induced seizures. Human reference *DMAP1* could largely compensate for the loss of *dDMAP1* in knockdown flies, whereas patient variants failed to restore or differentially rescued the phenotypes, confirming their pathogenicity with differing severity. Transcriptome profiling of *dDMAP1*-knockdown fly brains nominated Cbl and SF1 as downstream targets. Their overexpression rescued the aforementioned lethality and MB defects. Finally, a DNA methylation episignature was identified, leading to the molecular diagnosis of an additional patient. Our findings demonstrate that biallelic inactivating variants in *DMAP1* cause a syndromic NDD, expanding the short list of recessive disease-causing genes within the epigenetic machinery.

## Abstract 2

*DMAP1* variants cause a syndromic neurodevelopmental disorder

## Introduction

DNA methyltransferase 1–associated protein 1 (DMAP1) is encoded by *DMAP1* and participates in a multitude of functions, including DNA methylation, histone acetylation, histone deposition, and DNA damage repair (1–4). DMAP1 plays a critical role in the regulation of gene expression through its involvement in DNA methylation by interacting with DNA methyltransferase 1 (DNMT1) via its coiled-coil domain (4). This helps stabilize and target DNMT1 to replication foci, thereby aiding in the maintenance of DNA methylation patterns during DNA replication. This is essential for preserving epigenetic information and ensuring cellular identity in dividing cells. DMAP1 is also a scaffolding protein incorporated into the TIP60/TRRAP complex, which is a histone acetyltransferase complex involved in chromatin remodeling, DNA damage repair, and transcriptional regulation (5–8). These interactions mainly activate transcription by enhancing histone acetylation and facilitate DNA damage repair. Moreover, DMAP1 participates in the catalysis of exchanging histone H2A and H2A.Z within the SRCAP complex to regulate gene expression (9). Through these varied interactions, DMAP1 contributes to the modification of chromatin structure, the dynamic switching between transcriptionally inactive and active states, and the maintenance of genomic stability. This function is crucial in the context of development and cellular differentiation. Overall, DMAP1 is a multifunctional protein that plays a fundamental role in epigenetic regulation and cellular homeostasis.

Pathogenic variants in genes encoding components of these complexes have been described in neurodevelopmental and neurodegenerative disorders. These include *DNMT1* in autosomal dominant cerebellar ataxia, deafness, and narcolepsy (Mendelian Inheritance in Man [MIM]: 604121) (10); *SRCAP* and *KAT5*, encoding catalytic subunits of the SRCAP and TIP60 complexes, respectively, in Floating-Harbor syndrome (MIM: 136140) (11), developmental delay, hypotonia, musculoskeletal defects, behavioral abnormalities (MIM: 619595) (12), and a neurodevelopmental disorder (NDD) with dysmorphic facies, sleep disturbance, and brain abnormalities (MIM: 619103) (13); and *TRRAP* and *ACTL6A*, encoding scaffolding proteins, in developmental delay with or without dysmorphic facies and autism (MIM: 618454) (14) and intellectual disability (ID) (15), respectively. Many of these genes and gene complexes directly impact DNA methylation. DNA methylation, a key epigenetic regulator of gene expression, exhibits stable and tissue-specific patterns (16–20). In recent years, an increasing number of episignatures have been utilized as stable and reliable biomarkers for the diagnosis of congenital genetic disorders and for the reclassification of variants of uncertain significance (17–20), as have been implemented in clinical diagnostic laboratories with demonstrated diagnostic utility in genetically unresolved patients with suspected rare disorders (16).

*Drosophilamelanogaster* DMAP1 (dDMAP1 for the purpose of this study) is required for both larval and pupal development, the innate immune response (21), and chromosome segregation and cytokinesis during male meiosis (22). A critical role for Dmap1 in mammalian development has also been demonstrated in mice, where knockdown of *Dmap1* in mouse embryonic stem cells leads to a flattened and elongated cell morphology, reduced cell-cell contact and growth in a monolayer, and a reduction in proliferation (23), and knockout embryos die during preimplantation stages (24).

Despite DMAP1’s essential roles in multiple transcriptional processes, a specific syndrome associated with DMAP1 rare germline variants has not yet been described. Here, integrating clinical phenotyping, exome/genome sequencing, modeling in fruit flies, transcriptomics, and methylomics, we delineated DMAP1’s role in neurodevelopment and behavior, validated the pathogenicity of *DMAP1* variants in an NDD, and identified potential downstream targets for future therapeutic development. We identified 20 previously undiagnosed individuals harboring biallelic variants in *DMAP1*. These patients displayed a host of heterogeneous neurodevelopmental phenotypes, including global developmental delay, ID, autistic features, seizures, hypotonia, feeding difficulty, and craniofacial dysmorphic features. We found that flies with dDMAP1 loss of function (LoF) exhibited defects in survival, neural morphology, and behaviors, which were rescued by expressing the human DMAP1 reference sequence (DMAP1^Ref^). While missense variants also rescued survival, they were defective in the neural behaviors exhibiting differential severity. Transcriptome profiling of fly brains deficient of dDMAP1 uncovered downstream effectors, including Cbl proto-oncogene (Cbl), which encodes an E3 ubiquitin ligase that regulates signal transduction, cell proliferation, and apoptosis by controlling the activity of RTKs (25–27). Overexpression of the downstream effectors partially rescued the neural defects in *dDMAP1*-deficient flies. Using genome-wide DNA methylation analysis, we identified a distinct methylation episignature. Thus, by integrating fly and human genetic studies, our work has identified a critical network of genes associated with NDDs, impinging on chromatin regulation, which serves as an evolutionarily preserved focal point for regulating human neurodevelopment and function.

## Results

### Identification of biallelic variants in DMAP1 in probands with neurodevelopmental phenotypes.

**Figure 1:**
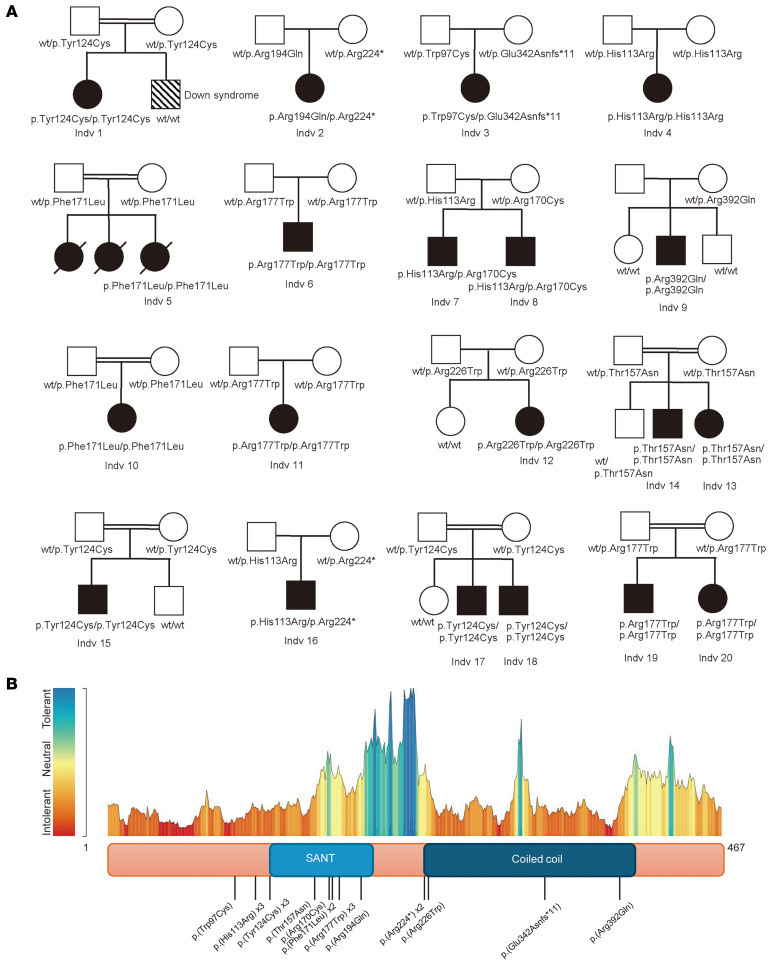
Molecular genetic findings in DMAP1 and facial photographs of affected individuals. (**A**) Pedigrees of studied families and cosegregating pattern of the *DMAP1* variants. The pedigrees are drawn using open circles to denote unaffected female subjects and open squares to denote unaffected male subjects; the solid shapes indicate affected subjects. (**B**) An intolerance landscape plot generated by MetaDome for *DMAP1* variant (NM_019100.5) analysis (top panel) and a schematic outline of the DMAP1 protein domains (lower panel) showing 5 variants observed in 2 or 3 families.

As an expansion of our study in identification of genetic causes of rare NDDs in an underrepresented cohort from the West Indies (28), exome analysis of a consanguineous family revealed a homozygous variant, c.371A>G, p.(Tyr124Cys), in *DMAP1* (NM_019100.5) in the proband affected with developmental delay, moderate ID, mild hypotonia, and failure to thrive. Subsequent Sanger sequencing confirmed the variant and showed that both parents are carriers. Through international collaboration, including via GeneMatcher, 20 individuals from 16 independent families with biallelic variants (either compound heterozygous or homozygous) in *DMAP1*, including 7 consanguineous families, have now been identified through exome sequencing, genome sequencing, or methylation analysis (Figure 1A). Individual (Indv) 5 was previously described in a large-scale cohort study (29). The variant spectrum included 10 missense, 1 nonsense, and 1 frameshift variants. Four missense variants [p.(His113Arg), p.(Tyr124Cys), p.(Phe171Leu), and p.(Arg177Trp)] as well as a nonsense [p.(Arg224*)] variant were detected in 2 or 3 independent families (Figure 1B and Supplemental Table 1; supplemental material available online with this article; https://doi.org/10.1172/JCI198229DS1). All identified variants were rare — either absent or with a minor allele frequency < 0.005% — in gnomAD v2. All missense variants were predicted to be deleterious by multiple bioinformatic algorithms (i.e., SIFT, PolyPhen2, Combined Annotation-Dependent Depletion, and M-CAP) and by three-dimensional structure modeling (Supplemental Table 1). Additionally, co-occurrence analysis within gnomAD v2 did not identify any individuals carrying the compound heterozygous variant combinations reported in this study, further supporting their pathogenicity. Notably, 8 out of 10 missense variants cluster around the SANT domain, which is responsible for facilitating interactions with DNA and histones, suggesting that these variants may affect these processes. All affected individuals presented with a similar core set of clinical features, which are summarized in Table 1 and described in more detail in Supplemental Table 1. All individuals have developmental delay involving speech and language, cognition, and motor skills. All individuals, except 2 cases that were too young for ID assessment, have ID ranging from mild to severe. Hypotonia was identified in 15 of 20 individuals (75%). Brain MRI scans were performed for 10 individuals and revealed malformations in 6 individuals (60%), including hypoplasia/agenesis of corpus callosum, dilated/asymmetrical ventricles, delayed myelination, and brain atrophy. Generalized and/or focal seizures occurred in 8 out of 20 individuals (40%). Behavioral problems were observed in 6 individuals (6/18), with 3 carrying a formal diagnosis of autism and 4 with attention deficit disorder/attention deficit hyperactivity disorder. Other notable findings include failure to thrive (11/20), microcephaly (8/20), short stature (4/20), and vision abnormalities (7/16). Clinical photographs of 11 individuals (including 2 sets of siblings: Indv 13 and 14, and Indv 17 and 18) show several shared craniofacial features, including large or broad forehead, high anterior hairline, elongated face, broad or depressed nasal bridge, upturned or small nose, flat philtrum, thin upper lip, open mouth appearance, and sparse hair. Moreover, GestaltMatcher analysis (Supplemental Figure 1A) showed that 83% of *DMAP1* distribution was below the threshold, indicating that the *DMAP1* patients share a similar facial phenotype. The 7 individuals (Indv 1, 6, 9, 13, 14, 17, and 18) are relatively similar in forming a cluster compared with the 3 other individuals (Indv 3, 11, and 12; Supplemental Figure 1B).

### dDMAP1 is essential for fly development, and patient DMAP1 variants exhibit partial LoF.

**Figure 2:**
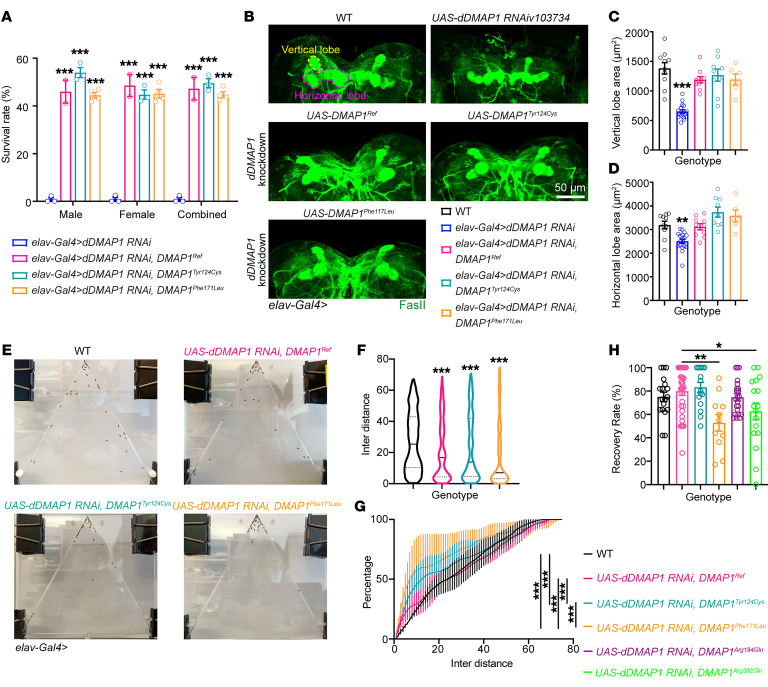
Loss of dDMAP1 leads to morphological and functional abnormality, and patient-derived DMAP1 variants differentially rescue developmental, structural, and behavioral phenotypes observed in *dDMAP1*-knockdown flies. (**A**) Pan-neural *dDMAP1* deficiency results in lethality in larvae. The expression of DMAP1^Ref^ or patient variants rescues lethality and significantly increases the survival rate. Analyzed by 2-way ANOVA followed by Tukey’s multiple-comparison test. *n* = 3, 2, 3, and 4 groups, with 30–118 male flies and/or 23–109 female flies in each group. (**B**–**D**) Pan-neuronal *dDMAP1* knockdown leads to structural defects of the MB in larval brains. The vertical MB lobe is dramatically thinner. The vertical MB lobe is outlined by the yellow dotted line, and the horizontal MB lobe is highlighted by the dotted magenta line. Scale bar: 50 μm. Expressing DMAP1^Ref^ or either of the patient missense variants rescues the volume of both the vertical (**C**) and horizontal (**D**) MB lobes to WT level. *n =* 10, 18, 10, 9, and 6 brains. (**E**–**G**) Expressing DMAP1^Ref^ or patient variants concurrently with *dDMAP1* knockdown rescues social behavior in the social space assay to different extents. *n =* 3 groups with 28–41 flies in each group. (**F**) Quantification of fly inter-distance in the triangle chamber. Analyzed by 1-way ANOVA followed by Dunnett’s multiple-comparison test. (**G**) Distribution of fly inter-distance. Analyzed by 2-way ANOVA followed by Tukey’s multiple-comparison test. (**H**) Pan-neural knockdown of *dDMAP1* predisposes flies to bang sensitivity, which can be rescued by expressing DMAP1^Ref^, DMAP1^Tyr124Cys^, or DMAP1^Arg194Gln^, but not DMAP1^Phe171Leu^ or DMAP1^Arg392Gln^. *n =* 18, 33, 16, 11, 22, and 17 groups with 9–14 flies in each group. Analyzed by 1-way ANOVA followed by Dunnett’s multiple-comparison test. *P < 0.05, **P < 0.01, ***P < 0.001.

To assess the impact of patient variants on *DMAP1*, we used *Drosophila melanogaster* as our model. When expressing *dDMAP1* RNAi under the control of a pan-neural driver, *elav-Gal4*, almost no larvae survived to the adult stage, implicating that dDMAP1 is essential for fly development and survival (Figure 2A). Notably, expressing either reference (DMAP1^Ref^) or patient missense variants (DMAP1^Tyr124Cys^ or DMAP1^Phe171Leu^) of DMAP1 was sufficient to rescue this lethality (Figure 2A). Since ID was identified as a clinical phenotype in DMAP1 patients, we then focused on assaying the mushroom body (MB), a structure homologous to the mammalian hippocampus that is recognized as the learning and memory center of flies. We found that pan-neural *dDMAP1* knockdown caused severe morphological defects in the MB, including decreased overall volume of the MB indicated by decreases in vertical and horizontal lobe area (Figure 2, B–D). The MB defect was replicated by a second independent *dDMAP1* RNAi, which showed a milder phenotype (Supplemental Figure 2, A–C). Again, expressing either reference DMAP1 or the 2 patient missense variants of DMAP1 fully restored the decrease in MB volume back to WT levels (Figure 2, B–D). Furthermore, *dDMAP1* knockdown specifically in fly sensory neurons driven by *ppk-Gal4*, which labels the nociceptive class IV dendritic arborization neurons, significantly decreased overall dendritic branching and reduced dendrite complexity at the late larval stages (Supplemental Figure 2, D and E). In WT neurons, the total dendrite length continued to increase during larval development. However, *dDMAP1* knockdown impaired this developmental process, which led to defects in dendritic tiling between neighboring neurons (Supplemental Figure 2F). While both DMAP1^Tyr124Cys^ and DMAP1^Phe171Leu^ increased total dendrite length in *dDMAP1*-knockdown larvae at 96 hours after egg laying, neither fully rescued the phenotype to the extent observed with DMAP1^Ref^ (Supplemental Figure 2, G and H). This result is indicative of dDMAP1 playing a direct role in postmitotic neurons to shape neuronal morphology and circuitry.

To determine if the observed neural structural abnormality led to functional deficits, the flies were subjected to a series of behavioral assays. This is feasible as both reference DMAP1 and patient variants rescued the lethality in *dDMAP1* pan-neural knockdown flies, allowing us to first conduct a social space assay to test the effect of missense variants DMAP1^Tyr124Cys^ and DMAP1^Phe171Leu^ on social behavior. WT flies are distributed widely in the behavior chamber, with some clustering at the top given their negative geotaxis (Figure 2E). We found that virgin female flies expressing either of the 2 human DMAP1 missense variants (DMAP1^Tyr124Cys^ or DMAP1^Phe171Leu^) exhibited a reduction in social space and impaired social avoidance, as determined by the decreased inter-distance when compared with flies expressing DMAP1^Ref^, which were largely comparable with WT flies (Figure 2, E–G). DMAP1^Phe171Leu^ performed the most poorly. This result indicated that although the 2 *DMAP1* missense variants extended the lifespan of *dDMAP1*-knockdown flies, they were unable to fully restore its function in neural processing and in the regulation of higher-order social behavior. Thus, these results suggest that dDMAP1 is required for normal fly social behaviors, and biallelic *DMAP1* variants affect neurons regulating social functions and most likely cause neurophysiological defects.

To further confirm the pathogenicity of the missense variants and given the seizure and hypotonia symptoms seen in patients, we performed the bang sensitivity test (Supplemental Figure 3), which assesses susceptibility to mechanically induced seizures (30, 31). Two additional patient variants, *DMAP1^Arg194Gln^* and *DMAP1^Arg392Gln^*, were also examined. Whereas WT flies quickly corrected their posture and started climbing immediately after vortex, flies expressing DMAP1^Phe171Leu^ or DMAP1^Arg392Gln^ in the setting of pan-neural *dDMAP1* knockdown showed delayed recovery from the stimuli (Figure 2H). In comparison, flies expressing DMAP1^Ref^, DMAP1^Tyr124Cys^, or DMAP1^Arg194Gln^ recovered as soon as WT (Figure 2H). These data collectively demonstrate that *DMAP1^Tyr124Cys^*, *DMAP1^Phe171Leu^*, and *DMAP1^Arg392Gln^* are all partial LoF alleles of DMAP1, while DMAP1^Tyr124Cys^ retains more residual function in this paradigm. Consistently, individuals 1, 15, 17, and 18, who carried the homozygous *DMAP1^Tyr124Cys^* variant, exhibited relatively mild clinical phenotypes, and none of them presented with seizures. In contrast, the 3 patients carrying *DMAP1^Phe171Leu^* or *DMAP1^Arg392Gln^* had seizures. Notably, the patient carrying *DMAP1^Arg194Gln^* also harbored a nonsense variant (p.Arg224*) and presented with seizures. Given that the nonsense variant is predicted to result in a more severe LoF effect, it may contribute more prominently to the phenotype in this individual, making it difficult to attribute pathogenicity to *DMAP1^Arg194Gln^* independently based on the current data. Therefore, further functional studies are required to determine the role *of DMAP1^Arg194Gln^*.

### Transcriptome profiling and potential effectors downstream of dDMAP1.

**Figure 3:**
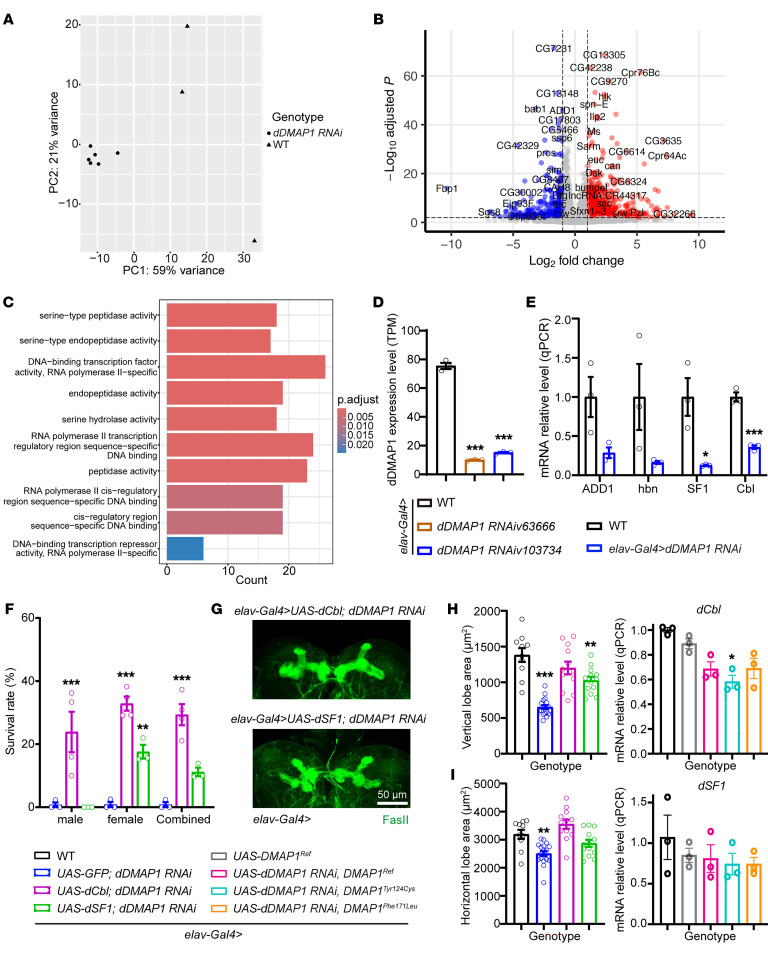
dCbl rescues the defects observed in *dDMAP1*-knockdown flies. (**A**) Principal component analysis showing the separation of the WT versus *dDMAP1* RNAi samples. (**B**) Differential expression analysis for WT versus *dDMAP1* RNAi, with at least 2-fold change and an adjusted *P* value < 0.01 (FDR corrected). (**C**) Gene Ontology enrichment analysis of downregulated genes shows significant enrichment for molecular functions related to DNA- and RNA-binding processes. (**D**) *dDMAP1* expression data in the brains of WT and 2 RNAi strains measured by transcripts per million (TPM). *n =* 3. Analyzed by 1-way ANOVA followed by Dunnett’s multiple-comparison test. ***P < 0.001. (**E**) qPCR data show that *dSF1* and *dCbl* are substantially downregulated in pan-neuronal *dDMAP1*-knockdown larval brains. Analyzed by unpaired 2-tailed Student’s *t* test. *n =* 3. ***P < 0.001. (**F**) Expressing dCbl pan-neuronally drastically increases the survival rate of *dDMAP1*-knockdown flies, while dSF1 partially rescues the lethality in female flies. Analyzed by 2-way ANOVA followed by Tukey’s multiple-comparison test. *n* = 3, 4, and 3 groups, with 18–43 male flies and/or 30–50 female flies in each group. (**G**–**I**) Expressing dCbl or dSF1 significantly attenuates the reduced MB area caused by *dDMAP1* deficiency. Scale bar: 50 μm. (**H**) Quantification of MB vertical lobe area. (**I**) Quantification of horizontal lobe area. *n =* 10, 18, 12, and 13 brains. (**J**) qPCR data show that *DMAP1^Ref^*, *DMAP1^Tyr124Cys^*, and *DMAP1^Phe171Leu^* similarly rescue the *dCbl* and *dSF1* levels in the pan-neural *dDMAP1* RNAi background. Pan-neural *DMAP1^Ref^* overexpression does not increase *dCbl* and *dSF1* levels in the WT background. All data are compared with the WT. Analyzed by 1-way ANOVA followed by Dunnett’s multiple-comparison test. *n* = 3. *P < 0.05, **P < 0.01, ***P < 0.001.

Given the involvement of DMAP1 in multiple complexes to regulate gene expression, we performed RNA-seq on brain tissues harvested at the third instar larval stage from WT controls and *elav-Gal4*–driven *dDMAP1*-knockdown flies. A principal component analysis plot supported the separation of the WT versus *dDMAP1* RNAi samples (Figure 3A). Differential expression analysis identified 1,182 genes with at least 2-fold change and an adjusted *P* value < 0.01 (FDR corrected), of which 873 were upregulated (73.9%) and 309 (26.1%) were downregulated (Figure 3B). Gene Ontology enrichment analysis of downregulated genes revealed significant enrichment for molecular functions related to DNA- and RNA-binding processes (Figure 3C), consistent with the known roles of DMAP1 in chromatin remodeling and transcriptional regulation. We focused on downregulated genes, as these are more likely to represent the direct consequences of impaired chromatin remodeling. *dDMAP1* RNAi efficiency was confirmed by an 82% reduction in the *dDMAP1* expression level (Figure 3D).

To nominate candidate downstream targets, downregulated fly genes were mapped to their human orthologs. Four genes were selected for validation based on their biological relevance, and reduced expression of all 4 was confirmed in *dDMAP1-*knockdown brains by qPCR (Figure 3E). Interestingly, human orthologs of 2 of these genes (*Cbl* and *SF1*) with high ortholog scores (10/14 and 13/14, respectively), are either known NDD-causing or NDD candidate genes: *CBL* in Noonan-like syndrome (32, 33) and *SF1* in NDD (34). To assess functional relevance, we expressed dCbl (*Drosophila* Cbl) or dSF1 (*Drosophila* SF1) in *dDMAP1-*knockdown flies under the control of *elav-Gal4* and found that dCbl drastically increased the survival rate in adult flies (Figure 3F). Interestingly, while dSF1 significantly attenuated the lethality in female flies, it had no effect in male flies. We then investigated the MB structure and found that expression of dCbl fully restored the volume of both vertical and horizontal lobes of MB, whereas dSF1 also partially rescued the morphological defects in *dDMAP1-*knockdown brains (Figure 3, G–I). Pan-neural overexpression of dCbl or dSF1 in the WT background did not result in increased overall brain size or MB lobe size (Supplemental Figure 4), suggesting that the rescue effects reflect compensation for the functional loss of dDMAP1 rather than a nonspecific increase in brain or MB volume. These results suggest that dCbl and dSF1 are the potential downstream effectors of dDMAP1. Furthermore, we found that *DMAP1^Ref^*, *DMAP1^Tyr124Cys^*, and *DMAP1^Phe171Leu^* similarly rescued the *dCbl* and *dSF1* levels in the pan-neural *dDMAP1*-knockdown background (Figure 3J). These data are consistent with our result that they were able to rescue the MB phenotype. Overexpression of *DMAP1^Ref^* in the WT background did not increase the *dCbl* and *dSF1* levels (Figure 3J), suggesting a possible regulatory mechanism keeping the expression of *Cbl* and *SF1* in check.

### Episignature analysis.

Given the critical role of DMAP1 in DNA methylation, we performed an episignature analysis using blood-derived genomic DNA from 8 individuals harboring *DMAP1* variants. This analysis identified 214 differentially methylated CpG probes that reliably distinguished affected individuals from 56 age-, sex-, and array-matched controls. The specificity and robustness of these probes were evaluated using hierarchical clustering and multidimensional scaling (MDS) analyses (Supplemental Figure 5), which demonstrated clear separation between DMAP1 cases and controls. To assess the reproducibility of the episignature, we performed leave-one-out cross-validation (LOOCV), in which 1 case sample was excluded per iteration during probe selection and subsequently reintroduced for testing and visualization via MDS. As illustrated in Supplemental Figure 6, most withheld samples consistently clustered with the remaining DMAP1 cases, confirming the reproducibility of the identified episignature. Support vector machine (SVM) models were then generated based on the 214 CpG probes to compute methylation variant pathogenicity (MVP) scores ranging from 0 to 1, with higher values reflecting greater similarity to the DMAP1-specific episignature. Two complementary models were developed as part of the standard EpiSign workflow. The first model (Supplemental Figure 7) was trained on the 8 *DMAP1* cases and the 56 age- and sex-matched controls. This baseline model is routinely used to assess the specificity of the episignature relative to the broader EpiSign Knowledge Database (EKD). While a small number of non-DMAP1 EKD samples displayed modestly elevated MVP scores, the vast majority scored near zero, demonstrating strong baseline specificity and no evidence of appreciable overlap with other episignatures.

**Figure 4:**
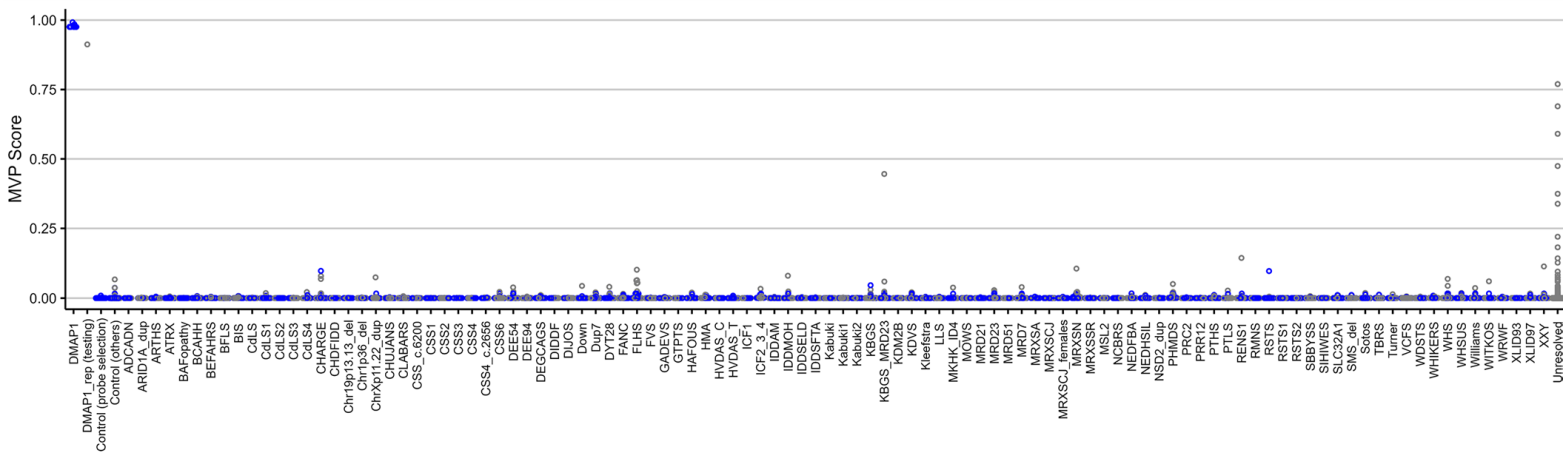
Classification model trained using matched controls and other disease samples. MVP scores generated by the SVM model, which is constructed by training the affected individual samples against matched control samples, 75% of other control samples from the EKD, and 75% of samples from other rare genetic disorders from the previously published EpiSign v5 classifier within the EKD. The remaining 25% of samples were used for testing the model. Samples used for training are shown in blue, and testing samples are shown in gray. The 56 matched controls are labeled “Control (probe selection),” whereas the remaining controls in the database are labeled “Control (others).” The “Unresolved” samples represent those that did not match any of the existing episignatures and may have unknown genotype or phenotype information. A list of episignature disorders is provided in Supplemental Table 2.

**Figure 5:**
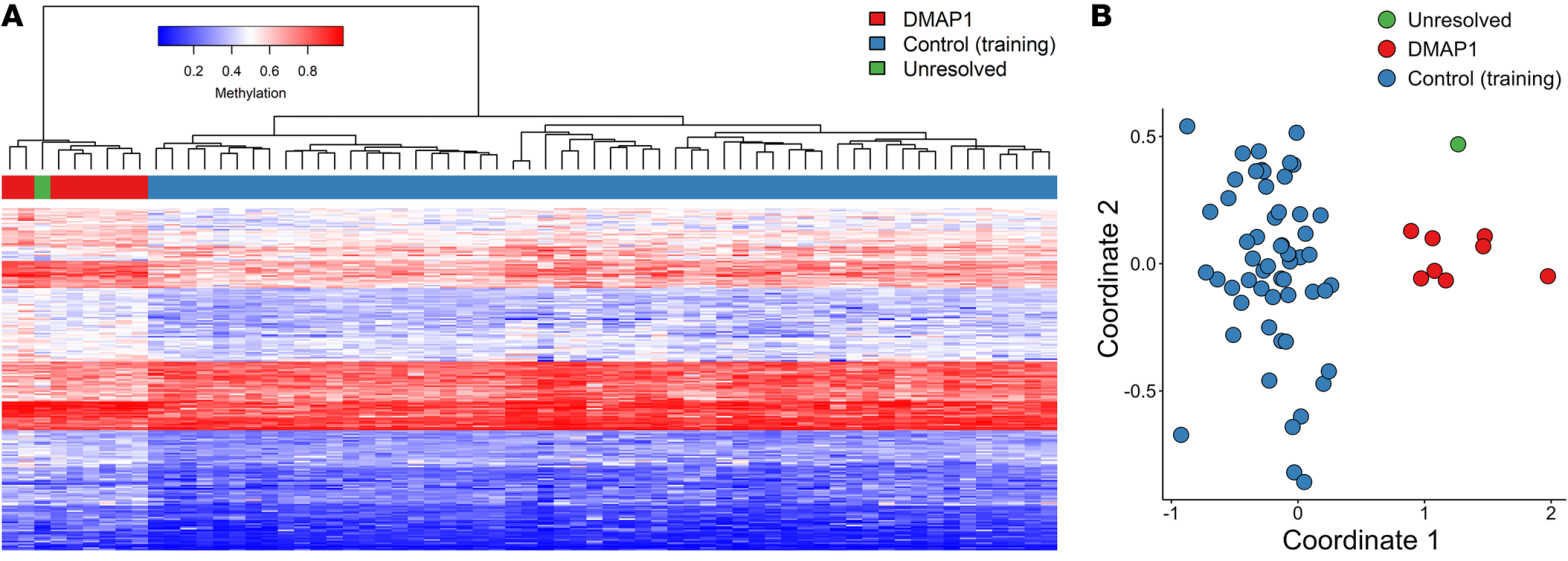
Identified episignature enabled identification of DMAP1 variants in a previously unresolved individual. (**A**) Hierarchical clustering heatmap, with rows representing the selected probes and columns representing individual samples. Methylation levels, ranging from 0 (unmethylated) to 1 (fully methylated), are shown using a blue-to-red color scale. In the heatmap panel, red denotes DMAP1 samples, green indicates the previously unresolved individual, and blue represents matched controls. (**B**) MDS plot using the same color scheme as in **A**, showing clear separation of cases and controls and the clustering of the unresolved samples with the cases.

A second model was then constructed to refine the specificity of the *DMAP1* episignature by incorporating 75% of additional control and disease samples from the EpiSign v5 clinical classifier into the training set. Results from this expanded model are shown in Figure 4. As expected, inclusion of the full spectrum of comparative episignature further increased specificity and robustness of the classifier. The final column of Figure 4 shows MVP scores for thousands of unresolved clinical cases submitted for diagnostic evaluation. Many of these samples lacked definitive genetic findings or detailed phenotypic information. Notably, follow-up investigation at the submitting institution confirmed that 1 individual with an elevated MVP score (Indv 16, MVP score: 0.59; Supplemental Table 1) carried biallelic pathogenic *DMAP1* variants. Clustering results for this individual are shown in Figure 5, A and B.

## Discussion

Here, we describe a syndromic NDD caused by biallelic inactivating variants in *DMAP1*, a gene encoding a versatile protein that participates in several distinct complexes in the repression or activation of transcription. Pathogenic variants were identified in 20 individuals from 16 unrelated families. Affected individuals exhibit a consistent spectrum of clinical features, primarily involving neurodevelopment and craniofacial morphology. Based on our findings, individuals harboring biallelic pathogenic *DMAP1* variants should undergo comprehensive clinical evaluation, as they are at risk of ID, structural brain abnormalities, seizures, short stature, cardiac anomalies, and neurobehavioral challenges.

Neurodevelopment is governed by both genetic and epigenetic mechanisms. Recent work has highlighted the prominent roles of chromatin structure or state in mediating brain development and diseases, which can be altered by chromatin remodelers via 3 distinct mechanisms (35). More specifically, chromatin remodelers accompany the development, migration, and circuit formation of major cortical cell types: glutamatergic and GABAergic neurons and glia. Consequently, dysregulation of chromatin remodeling may impact the development of each of these cell types, resulting in lasting impairments in brain wiring and function (35). There are 4 main families of ATP-dependent chromatin remodeling complexes categorized based on their unique domains and associated subunits: SWI/SNF (switch/sucrose-nonfermenting), ISWI (imitation switch), CHD (chromodomain-helicase-DNA binding), and INO80 (inositol requiring 80). DMAP1 belongs to the multisubunit INO80 subfamily, which remodels chromatin by either sliding nucleosomes along the DNA or exchanging histones within nucleosomes, playing crucial roles in DNA repair, replication, transcription, and histone exchange (36–38). While numerous NDD-linked genes have been reported in the SWI/SNF, ISWI,and CHD complexes, pathogenic variants in the INO80 subfamily components are relatively scarce, with SRCAP (11, 12) and YY1AP1 (39, 40) having been nominated so far. Our study provides functional evidence that implicates *DMAP1* variants as a cause of NDD.

To date, more than 100 human developmental disorders have been attributed to germline variants in genes encoding components of the epigenetic machinery (41–44). The majority of these disorders exhibit an autosomal dominant or X-linked mode of inheritance (41–44), whereas only a few have an autosomal recessive pattern, including *ERCC6* (45, 46), *ACTL6B* (47), *KDM5B* (48), *TASP1* (49), *HELLS* (50), *CDCA7* (50), and *DNMT3B* (51). Our findings extend the list of recessive causative genes implicated in epigenetic machinery.

Notably, the neurodevelopmental deficits associated with the *DMAP1* variants identified to date appear to fall on the milder end of the NDD spectrum. This is reflected by the full rescue of the fly survival and MB morphological defects by the 2 missense variants tested, Tyr124Cys and Phe171Leu, with pathogenicity manifesting only in higher-order cognitive assays (Figure 2, E–H). Remarkably, the Tyr124Cys and Arg194Gln variants were able to rescue the bang sensitivity in flies, whereas Phe171Leu or Arg392Gln did not (Figure 2H). This functional divergence aligns with our patient phenotypes: 4 individuals (1, 15, 17, and 18) homozygous for Tyr124Cys had not developed seizures by ages 7, 4, 13, and 7, respectively, while 3 other individuals (5, 10, and 9) homozygous for Phe171Leu or Arg392Gln developed seizures at 1, 2, and 0.5 years of age, respectively. These findings support a genotype–phenotype correlation and underscore the value of functional assays in stratifying variant pathogenicity, which reflect not only gross morphology but also synapse formation, neuronal physiology, and network-level connectivity. Therefore, while different experimental readouts can differ considerably in their sensitivity to mutational effects, behavioral phenotypes may be better able to discriminate among different missense variants, including those that produce only subtle or partially overlapping structural defects. However, additional individuals and further assessment will be required to validate this correlation. Although our functional experiments demonstrate LoF effects for our missense *DMAP1* variants, the degree of residual function appears to vary; therefore, the function of each variant needs to be carefully analyzed.

Functional integrations among chromatin remodelers and their regulatory proteins have been shown to converge on shared transcriptional axes (35). As an effort to delineate the genetic network of NDD-associated factors, we have begun to uncover key nodes downstream of various transcription regulatory modalities. In the case of chromatin remodelers, we identified 2 downstream effectors for DMAP1: SF1 and Cbl. *SF1* encodes Splicing Factor 1, a pre-mRNA splicing factor involved in the ATP-dependent formation of the spliceosome complex. A recent study identified *SF1* variants as the cause of a NDD with variable severity and autistic traits (34). *Cbl* encodes a RING finger E3 ubiquitin ligase that interacts with activated RTKs, upstream of PI3K and RAS, leading to ubiquitylation and degradation of the RTKs. Pathogenic variants in *CBL* have been implicated in Noonan syndrome–like disorder (32, 33), characterized by features including ID, developmental delay, and cardiac defects. Notably, several of these features overlap with the clinical phenotype observed in individuals with biallelic DMAP1 variants, who similarly present with ID, developmental delay, and, in a subset of patients, structural brain anomalies and seizures. This phenotypic convergence between DMAP1- and CBL-associated NDDs lends further support to a functional link between these 2 genes and raises the possibility that dysregulation of the DMAP1/CBL axis may contribute to shared disease mechanisms across this phenotypic spectrum. Reduced expression of Cbl would lead to impaired ubiquitin ligase activity, in turn leading to upregulation of RAS/MAPK signaling. These findings suggest that further investigation of the DMAP1/CBL regulatory axis may offer therapeutic opportunities, including the potential repurposing of RTKs or downstream ERK/MEK inhibitors. More broadly, these findings emphasize the importance of identifying converging molecular pathways to inform future therapeutic interventions. However, due to the differences in species, tissue type, and developmental context, a direct correlation between DMAP1-associated methylation changes and altered expression of Cbl or SF1 in the fly model could not be established. Future studies using patient-derived neuronal cells or isogenic human neural models will be better suited to determine whether and how DMAP1-mediated differentially methylated CpGs directly regulate target gene expression.

Using genome-wide DNA methylation analysis on 8 individuals, we identified a distinct methylation episignature. This episignature successfully classified a blinded, unresolved subject (Indv 16; Figure 5), demonstrating its potential as a sensitive and specific biomarker to be used as a diagnostic test to identify unresolved patients with *DMAP1*-related NDD. However, due to the limited cohort size, LOOCV did not yield optimal results for all samples. Ongoing work focused on increasing the number of cases in the reference cohort is expected to further improve the performance of the classifier model.

Given the versatile functions of DMAP1 in transcriptional regulation and its diverse interacting partners, future studies comparing its role with that of its cofactors in distinct complexes will be critical for pinpointing the exact mechanisms of pathogenesis underlying specific variants and their associated syndromic symptoms. A key question is how mutations in broadly acting epigenetic machinery, such as chromatin remodelers, lead to relatively specific defects in the nervous system. We postulate that the answer may lie in the locus, complex composition, temporal, and cell-type specificity. Chromatin modifications are site specific at the genomic level, and the chromatin remodeler complexes are dynamically assembled and disassembled based on location and time. Determining which genes are modified by which remodelers, at specific time points and in defined cell types, and developing genetic tools to manipulate these modifications with spatiotemporal precision, are key to eventually deciphering the epigenetic code.

## Methods

### Sex as a biological variable.

Sex was not considered as a biological variable in this study. For the human cohort, all individuals with confirmed pathogenic or likely pathogenic *DMAP1* variants were included regardless of sex. For most *Drosophila* experiments, both male and female larvae or adult flies were used. The only exception was the social space assay, where only virgin females were used to minimize variability associated with mating status, courtship, aggression, and sex-specific social interactions, ensuring a more consistent baseline for assessing genotype-dependent social spacing phenotypes.

### GestaltMatcher facial analysis.

We conducted a GestaltMatcher-based facial phenotype analysis on 8 individuals harboring pathogenic variants in the *DMAP1* gene. Individual 15 was excluded from this analysis due to an unsuitable facial angle for accurate GestaltMatcher processing. Individuals 13 and 14 belong to one family, and individuals 17 and 18 belong to another. All siblings were included to evaluate whether a consistent facial phenotypic pattern existed among *DMAP1* patients. The GestaltMatcher model (52) was trained on 8,547 facial images spanning 244 syndromes from GestaltMatcher Database (GMDB) (53), enabling the extraction of dysmorphic facial features. Each patient image was processed using test-time augmentation and model ensembling, resulting in 12 facial phenotype descriptors per image. Cosine distances between these facial phenotype descriptors were averaged to quantify facial similarity, with lower distances indicating higher resemblance.

We evaluated similarity both at the cohort and individual levels (54). For cohort-level analysis, we examined whether the facial similarity among *DMAP1* patients exceeded what is expected by chance. To do so, we first computed the mean pairwise cosine distances for the *DMAP1* cohort and compared this distribution to 2 controls generated from GMDB: (a) cohorts consisting of patients with the same disorder and (b) randomly sampled patient cohorts. Specifically, we randomly selected subsets of size *n* (2 ≤ *n* ≤ 8) from the *DMAP1* cohort and computed their mean pairwise distances. This sampling was repeated 100 times (removing duplicates), forming a distribution of within-cohort distances. A receiver operating characteristic (ROC) analysis, based on 5-fold cross-validation, was conducted using mean pairwise distances from known same-syndrome and random cohorts to derive a decision threshold. The optimal cut-off was *c* = 0.915, corresponding to a sensitivity of 0.851, a specificity of 0.862, and an AUC of 0.895. We then assessed what proportion of the *DMAP1* distribution fell below this threshold, thereby supporting phenotypic coherence within the group.

To assess individual-level similarity, we conducted a pairwise rank analysis. Using a leave-one-out approach, each *DMAP1* individual was compared against a background of 7,459 images across 449 syndromes in GMDB. For each test subject, the remaining 7 *DMAP1* individuals were embedded into this reference space, and their ranks were recorded based on cosine similarity to the test subject. This allowed us to quantify how closely *DMAP1* patients resemble each other compared with a broader syndromic patient group.

### Fly stocks.

The *w^1118^*, *UAS-dDMAP1 RNAiv103734*, *UAS-dDMAP1 RNAiBL63666*, *UAS-dCbl*, *UAS-dSF1*, *elav-Gal4*, *UAS-Dcr2*, and *UAS-mCD8:GFP* stocks were obtained from the Bloomington Drosophila Stock Center, FlyORF, or Vienna Drosophila Resource Center (VDRC). To generate the *UAS-DMAP1^Ref^*, *UAS-DMAP1^Tyr124Cys^*, *UAS-DMAP1^Arg194Gln^*, *UAS-DMAP1^Arg392Gln^*, and *UAS-DMAP1^Phe171Leu^* stocks, the coding sequences were synthesized (GenScript) and constructed into the pACU2 vector, then injected into fly embryos (Rainbow Transgenic Flies) using øC31-based attp40-specific insertion.

### RNA-seq and analysis.

RNA was extracted for all the fly brain tissue samples (3 WT and 6 RNAi) at the same time to minimize the batch effect. For each sample, approximately 30 WT or pan-neuronal knockdown larval brains were collected, with 3 biological replicates for each genotype. RNA was extracted with TRIzol (Ambion, 15596026). Extracted RNA samples underwent quality control assessment using Bioanalyzer (Agilent) and were quantified using NanoDrop Technologies. RNA libraries were prepared using the poly(A) method and were sequenced using the NovaSeq 6000 sequencer (Illumina) at the Center for Applied Genomics at the Children’s Hospital of Philadelphia per standard protocols (paired-end, 100 bp). The RNA-seq data were aligned on the BDGP6 fly using HISAT2 v2.1.0 (55), transcripts were assembled using StringTie v2.0, and features were counted by featureCounts. DESeq2 was then used to detect differentially expressed genes.

### RT-qPCR.

RT-qPCR was performed as previously described (56). RNA was extracted from fly brains with TRIzol, and reverse transcription was performed with a cDNA synthesis kit (Bio-Rad, 1708891). The mRNA levels of *Drosophila* *ADD1*, *hbn*, *Cbl*, and *SF1* were normalized to *rp49*. The mRNA levels of the tested genes in *dDMAP1*-knockdown brains were normalized to that in WT. The primers used are as follows: rp49 (F: CAGTCGGATCGATATGCTAAGCTG and R: TAACCGATGTTGGGCATCAGATAC), ADD1 (F: GAGCAAAACGAGAACTGGAAC and R: AAGCTGAAGGGTTTGTAGGG), hbn (F: ACCCAGTATCCCGATGTTTTC and R: GGCCTTGTCCTGATTCATAAAC), Cbl (F: GGCTATCGCTTCCTCTGTTT and R: AGAGGCGAACGTGATCAATAG), and SF1 (F: GAGGAGATTAGTCGCAAGCTG and R: TTCCAGTCTTTTCCTGTAGCG).

### Social space assay.

Social space assay was performed as described (57). In brief, 5–7 days after eclosion, approximately 40 virgin females were aspirated into the social space arena. The flies were given 20 minutes to freely investigate and acclimate to the arena and then images were captured. The distance between any of the 2 flies was measured by ImageJ (NIH) and normalized to the average body length of flies in pixels (58).

### Live imaging.

Embryos were collected for 2–24 hours on yeasted grape juice agar plates and were aged at 25°C. At 48 hours after egg laying, the plates were moved to room temperature. A single larva was mounted in 90% glycerol under coverslips sealed with grease, imaged using a Zeiss LSM 880 microscope, and returned to grape juice agar plates between imaging sessions. Neurons were reconstructed and analyzed with Neuronstudio (59).

### Immunohistochemistry.

Third instar larvae were dissected, and their brains were subjected to immunostaining with the mouse anti-FasII antibody (1:100, Developmental Studies Hybridoma Bank, 1D4) and fluorescence-conjugated secondary antibodies (1:1,000, Jackson ImmunoResearch).

### Bang sensitivity.

The bang sensitivity assay was performed as previously described (31), with minor modifications. Two days before the assay, adult flies were transferred into fresh vials in groups of approximately 10. On the day of the assay, approximately 10-day-old flies were flipped into empty vials without anesthesia. A horizontal line was drawn 3 cm from the base of each vial to serve as a climbing threshold and post-recovery indicator. Flies were then vortexed at speed 5 for 10 seconds. Following mechanical shock, recovery behavior was observed, and recovery time was recorded using https://www.timeanddate.com/stopwatch/ for stopwatch recording. Bang sensitivity was quantified as the latency for individual flies to correct their posture and climb above the 3 cm mark. Flies that failed to reach the 3 cm threshold within 60 seconds were assigned a recovery time of 60 seconds for analysis.

### DNA methylation episignature analysis.

DNA methylation profiling was approved by the Western University Research Ethics Board (REB 106302 and 116108) and performed using Illumina Infinium MethylationEPIC BeadChip microarrays, as previously described (17, 60). Genomic DNA extracted from peripheral blood of the case subjects was bisulfite converted and processed according to the manufacturer’s instructions. Raw signal intensities were imported into R (v4.4.1), and data were normalized using the SeSAMe package (v1.22.2) (61). Standard quality control filters were applied to exclude probes on sex chromosomes, SNP-associated sites, cross-reactive probes, and those flagged by Illumina as unreliable. Arrays with over 5% probe failure or previously identified in EKD (https://episign.lhsc.on.ca/index.html) as introducing batch effects were also excluded. Matched controls (*n* = 56; 7 per case) were selected from the EKD using the MatchIt package (v4.5.5; https://cran.r-project.org/web/packages/MatchIt/vignettes/MatchIt.html), ensuring matching based on age, sex, and array platform. Principal component analysis was used iteratively to remove outliers and confirm data structure. Methylation levels were quantified as the β-value, defined by the ratio of methylated signal intensity to the sum of methylated and unmethylated intensities. This value ranges from 0 (indicating no methylation) to 1 (indicating complete methylation). To improve statistical properties for analysis, β-values were converted to M-values using a logit transformation: log_2_(β/(1−β)). Differential methylation analysis was conducted using the limma package (version 3.58.1) (62), incorporating blood cell composition estimates (63) as covariates. *P* values were moderated with limma’s eBayes function and adjusted for multiple comparisons using the Benjamini-Hochberg procedure.

Episignature probe selection was performed through a 3-step process. First, 1,000 probes were chosen with the highest product of the average methylation difference between case and control groups and the negative log-transformed, adjusted *P* values from the linear model. Next, ROC analysis was applied, and the top 500 probes with the highest ROC AUC were retained. Lastly, probes with a pairwise Pearson’s correlation coefficient greater than 0.65 within either the case or control groups were excluded to reduce redundancy. This filtering process yielded a final set of 214 differentially methylated probes, which were then used to build a hierarchical clustering model using Ward’s linkage on Euclidean distance and an MDS model based on scaled pairwise Euclidean distances between samples. To evaluate the episignature reproducibility, LOOCV was performed across 8 separate rounds, with probe selection based on 7 cases and subsequent testing of the withheld sample via MDS clustering. A binary classification model using SVM was developed based on the 214 selected probes, utilizing the e1071 package as previously described (20, 60). The classifier assigns each sample an MVP score between 0 and 1, with scores closer to 1 indicating a methylation profile closely matching the syndrome-specific episignature. Two MVP plots were generated: 1 trained on 8 *DMAP1* cases and 56 matched controls and another incorporating 75% of additional control and disease samples from the EpiSign v5 clinical classifier for enhanced specificity. The remaining 25% of these samples were reserved for testing. This strategy allowed for the refinement of probe selection to minimize overlap with other disorders, thereby improving the specificity of the classifier. This 2-step approach enables both evaluation of potential overlap with other episignatures and generation of a final, highly specific disorder-specific classifier. The disorders included in the EpiSign v5 clinical classifier are provided in Supplemental Table 2.

### Statistics.

The data shown represent the results of at least 3 separate experiments. All results are presented as the mean ± SEM or the mean ± SD. Comparisons between 2 groups were analyzed by 2-tailed Student’s *t* test, and comparisons between 3 groups were done by 1-way ANOVA followed by Dunnett’s test or 2-way ANOVA followed by Tukey’s or Šidák’s test using GraphPad Prism (GraphPad Software). A *P* value of less than 0.05 was considered statistically significant.

### Study approval.

The protocol was approved by the Children’s Hospital of Philadelphia institutional review board, and informed consent was obtained as appropriate (IRB 16-013278). Written informed consent was also retained for all individuals whose photographs appear in the manuscript.

### Data availability.

RNA-seq data generated from flies are available in the NCBI’s Gene Expression Omnibus database (GSE326696). Values underlying the data presented in the graphs are provided in the Supporting Data Values file. Upon reasonable request, additional information can be provided from the corresponding author. Datasets used for DNA methylation analysis that are available publicly are previously described (20). Anonymized data for each subject are described in the study. The individual genomic and epigenomic or any other personally identifiable data for other samples in the EKD are prohibited from deposition in publicly accessible databases due to institutional and ethics restrictions. Specifically, these include data and samples submitted from external institutions to London Health Sciences EKD that are subject to Institutional Material and Data Transfer agreements, data submitted to London Health Sciences for episignature assessment under Research Services Agreements, and research study cohorts under Institutional Research Ethics Approval (Western University REB 106302 and 116108). Some of the software packages used in this study are publicly available as described in Methods. EpiSign is a commercial software and is not publicly available.

## Author contributions

YS and DL supervised the study. QW, CT, SH, MEM, LSM, HX, JZ, YC, MAL, J Kerkhof, HM, JR, CLY, BS, YS, and DL performed functional assessment and validation. AKS, TDH, KP, SR, MM, MLL, NC, AV, AP, NAAG, MEHS, AKY, DLA, RAS, FA, HES, NAMA, MA, VV, MJGS, IMW, NSD, RB, BVO, SMJH, MSZ, GE, EA, J Kim, SE, H Houlden, AN, MT, UA, ZI, ST, FSA, EJB, RM, H Hakonarson, YS, and DL conducted molecular and clinical evaluation of the affected individuals. TCH and JML conducted the GestaltMatcher analysis. QW, CT, YS, and DL wrote the original draft of the manuscript. All authors reviewed and edited the manuscript. The order of co–first authors was determined based on contribution, and a consensus agreement was reached to ensure mutual understanding and commitment.

## Conflict of interest

BS is a shareholder in EpiSign Inc., involved in commercial uses of EpiSign technology. MJGS and IMW are employees of and may own stock in GeneDx, and ST is an employee of Ambry Genetics.

## Funding support

- Eagles Autism Foundation grant (to DL).
- Children’s Hospital of Philadelphia Omics grant (to YS and DL).
- Government of Canada through Genome Canada and the Ontario Genomics Institute (OGI-188 to BS).
- Research, Development and Innovation Authority (RDIA), Kingdom of Saudi Arabia (award 12996-iau-2023-TAU-R-3-1-HW- to NAMA).
- Pakistan Science Foundation grant PSF/Res/P-AAU/Med (546) (to UA).

## Supplementary Material

Supplemental data

Supporting data values

## Figures and Tables

**Table 1:**
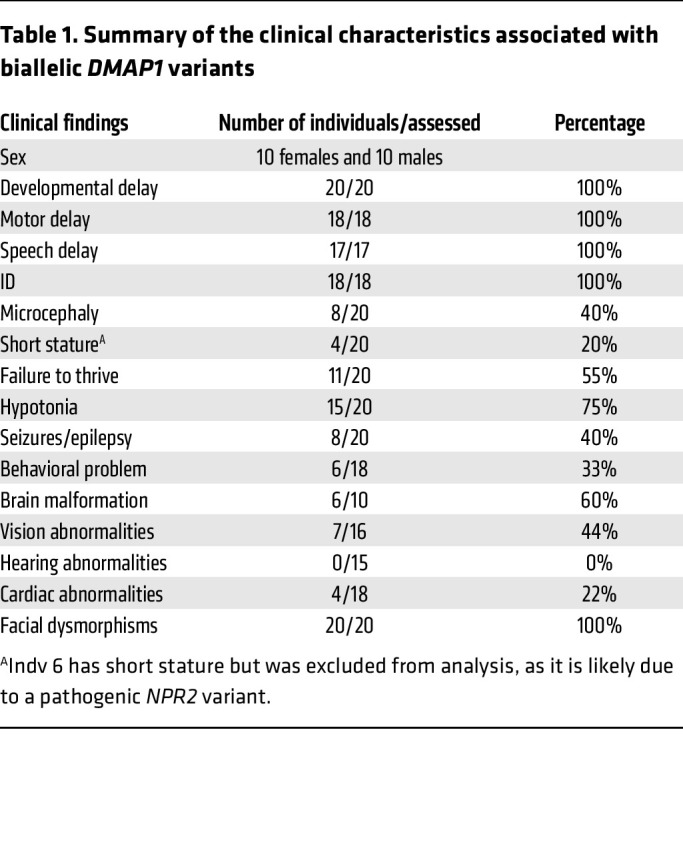
Summary of the clinical characteristics associated with biallelic *DMAP1* variants

## Version 1 06/11/2026

In-Press Preview

## Version 2 08/03/2026

Electronic publication
