# Supplementary material for: Biallelic inactivating variants in the chromatin remodeler DMAP1 cause a syndromic neurodevelopmental disorder
Source: J Clin Invest. 2026 Jun 11;136(15):e198229. doi: 10.1172/JCI198229 (PMC13430023; doi:10.1172/JCI198229)
Supplement: Supplemental data [file jci-136-198229-s141.pdf]

Supplementary Materials

A

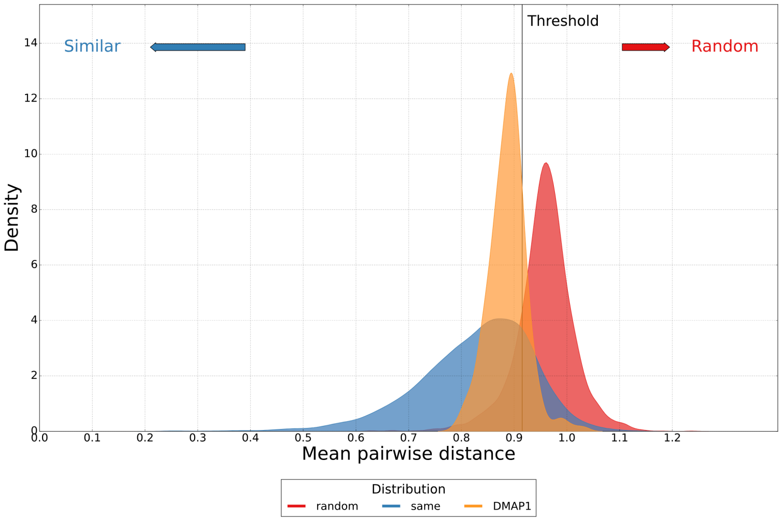

B

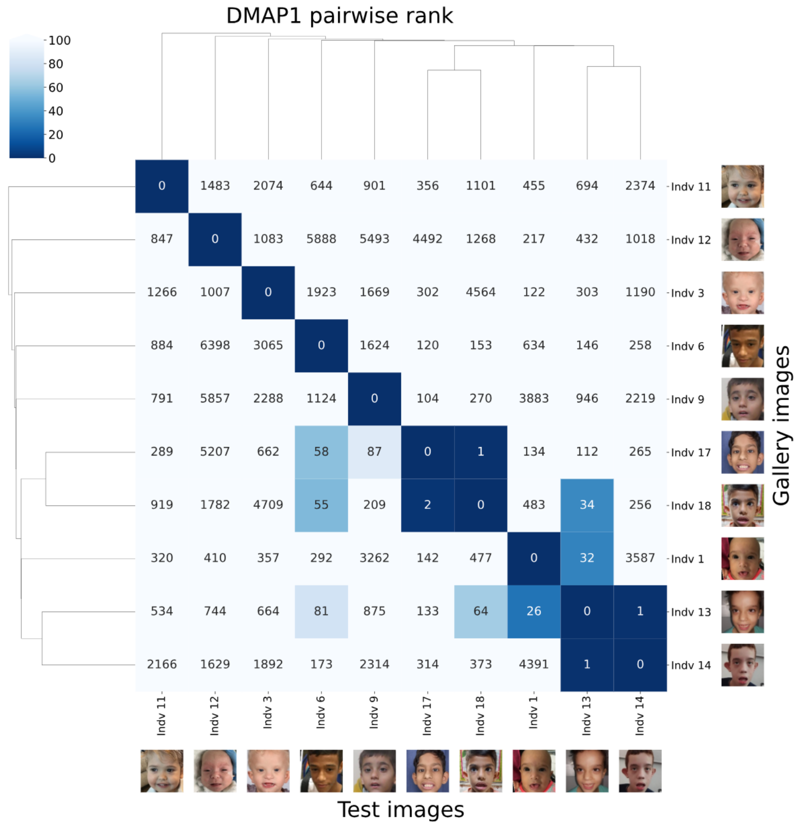

Supplemental Figure 1. Cohort level and pairwise facial similarity analysis of individuals with biallelic variants in *DMAP1*. (A) Kernel density estimation plot showing mean pairwise cosine distances for individuals with the same syndrome from Gestalt Matching Database (GMDB; blue), a randomly sampled cohort (red), and ten individuals with biallelic *DMAP1* variants (orange). The vertical black line marks the similarity threshold (c = 0.915) defined by ROC curve analysis. The DMAP1 cohort had a mean pairwise cosine distance of 0.874, with 83% of randomly drawn subsets falling above this threshold, supporting a recognizable shared facial gestalt. Lower cosine distance values indicate greater facial similarity. **(B)** Pairwise rank comparison among ten individuals with biallelic *DMAP1* variants. Each test image (columns) was compared against the remaining *DMAP1* individuals (rows) and GMDB images of individuals with other disorders. Cell values indicate the rank of each gallery image relative to all comparators for a given test image, with rank 1 indicating the greatest facial similarity. Siblings (Individuals 13 and 14) showed the strongest mutual similarity, each ranking the other as rank 1. The individual numbering corresponds to the identifiers listed in Supplemental Table 1.

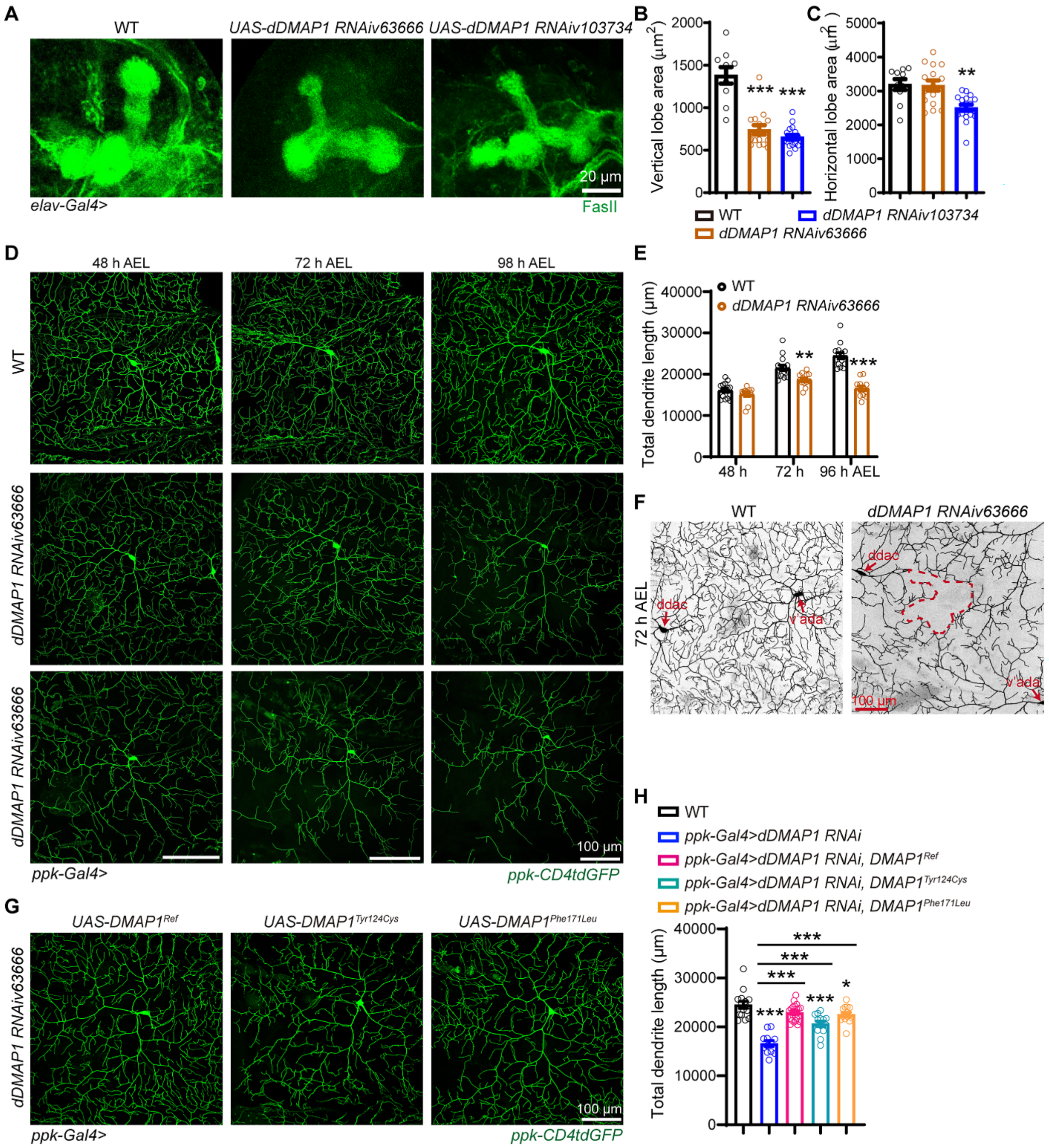

Supplemental Figure 2. Loss of dDMAP1 leads to mushroom body morphological and dendritic branching defects in flies. (A-C) Knocking down *dDMAP1* by two independent RNAis consistently leads to MB morphological defects. Scale bar, 20 μm. Quantification of MB area of vertical lobe **(B)** and horizontal lobe **(C)**, n = 10, 15, 18 brains. Analyzed by one-way

ANOVA followed by Dunnett's multiple comparisons test, ** $P < 0.01$, *** $P < 0.001$. **(D, E)** *dDMAP1* knockdown reduces total dendrite length at 72 h and 96 h AEL. Scale bar, 100 µm. n = 15, 12 neurons. Analyzed by two-way ANOVA followed by Šidák's multiple comparisons test, ** $P < 0.01$, *** $P < 0.001$. **(F)** *dDMAP1* knockdown causes tiling defects. (**G, H**) $DMAP1^{Ref}$ fully restored the total dendrite length in *dDMAP1* knockdown neurons at 96 h AEL, while the two missense variants only partially increased the dendrite length. n = 15, 12, 21, 13, 14 neurons. Analyzed by two-way ANOVA followed by Šidák's multiple comparisons test, * $P < 0.05$, *** $P < 0.001$. The 4 genotypes are compared to WT, asterisks without the line. $DMAP1^{Ref}$ is not different from the WT, whereas $DMAP1^{Tyr124Cys}$ and $DMAP1^{Phe171Leu}$ are statistically lower. The 3 rescue genotypes are also compared to the *dDMAP1 RNAi*, asterisks with the line. Scale bar, 100 µm.

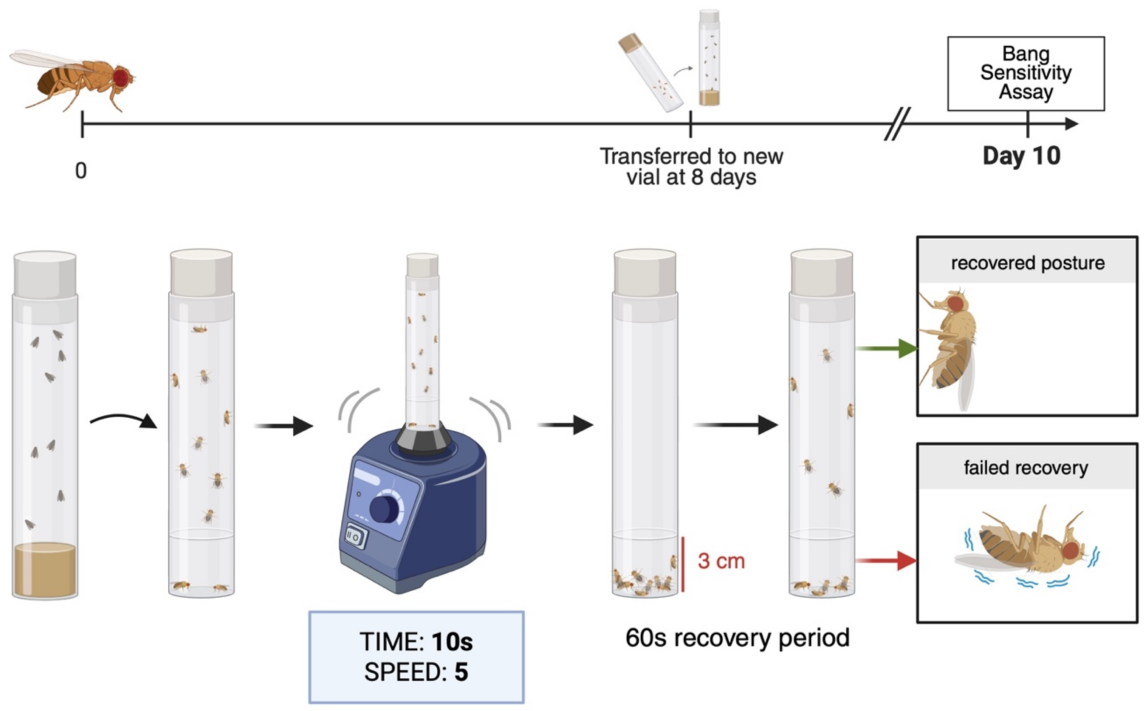

Supplemental Figure 3. Schematic of the Bang Sensitivity Assay in *Drosophila*. Flies were aged to 10 days post-eclosion. Flies were transferred to fresh vials without anesthesia on day 8, two days prior to testing. On the day of the assay, groups of ~10 flies were transferred without anesthesia into empty vials marked with a 3 cm line. Vials were vortexed at speed 5 for 10 seconds, then immediately placed upright for a 60-second recovery period. Bang sensitivity was quantified as the ability of individual flies to correct posture and climb above the 3 cm mark. Flies that successfully recovered were scored as "recovered posture," while those that remained incapacitated below the line were scored as "failed recovery."

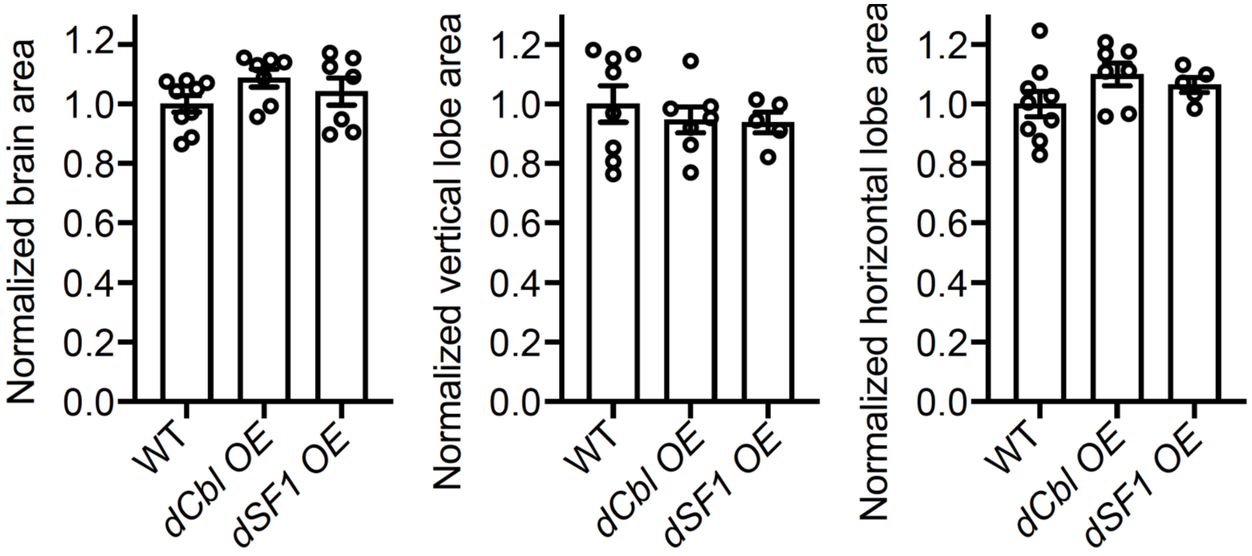

Supplemental Figure 4. Overexpression of dCbl and dSF1 does not increase brain or MB size. Pan-neural (*elav-Gal4*) *dCbl* or *dSF1* overexpression (OE) does not increase brain size in the WT background. Brain or MB lobe areas are normalized to that of the WT. Analyzed by one-way ANOVA followed by Dunnett's multiple comparisons test.

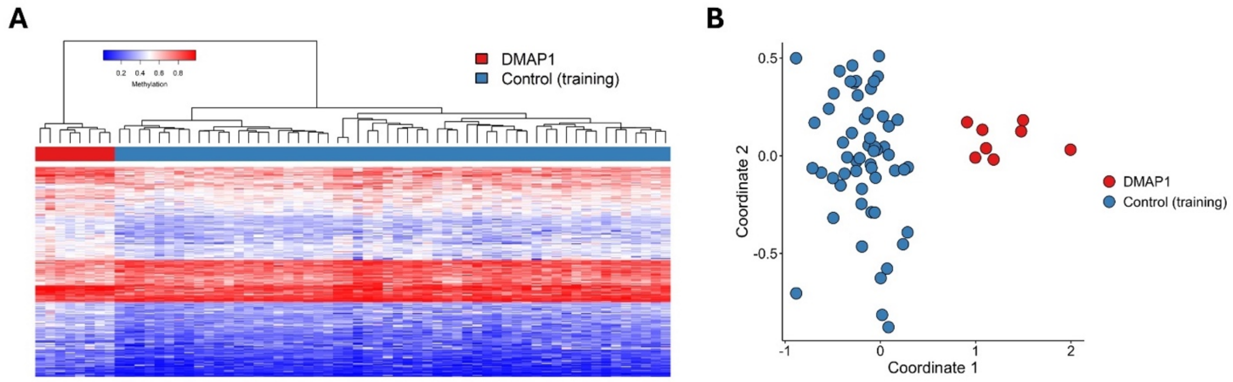

Supplemental Figure 5. Verification of the identified episignature. (A) Hierarchical clustering heatmap, with rows representing the selected probes and columns representing individual samples. Methylation levels, ranging from 0 (unmethylated) to 1 (fully methylated), are shown on a blue-to-red color scale. In the heatmap panel, red denotes DMAP1 samples and blue indicates matched controls. **(B)** MDS plot using the same color scheme as in panel A.

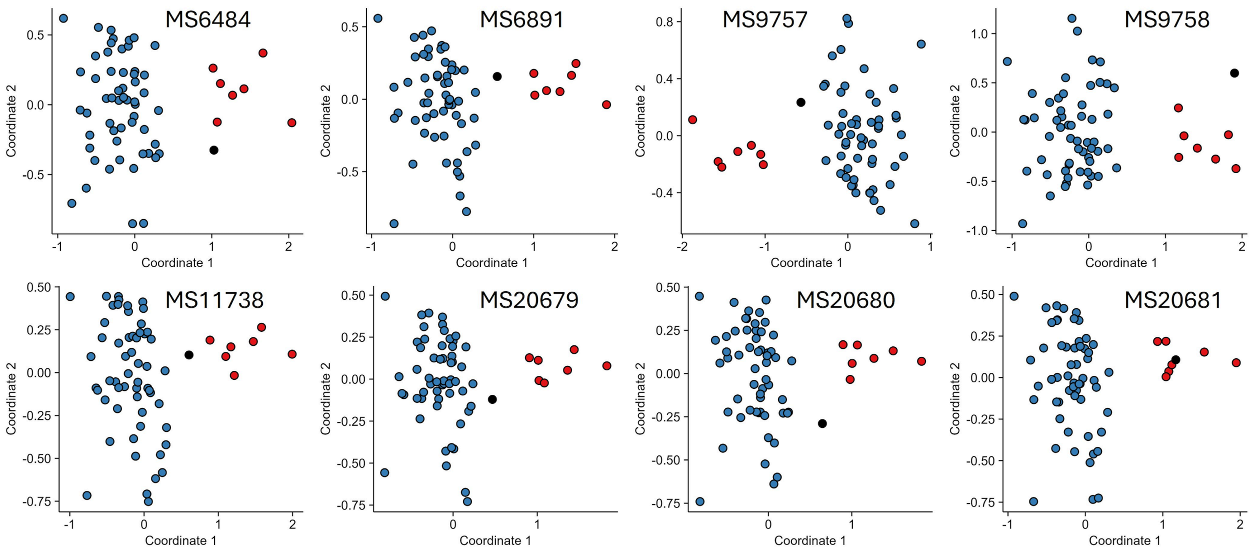

Supplemental Figure 6. Leave-one-out cross-validation. MDS plots generated for each round of leave-one-out cross-validation. Case and control samples used for probe selection are shown in red and blue, respectively, while the test sample is shown in black.

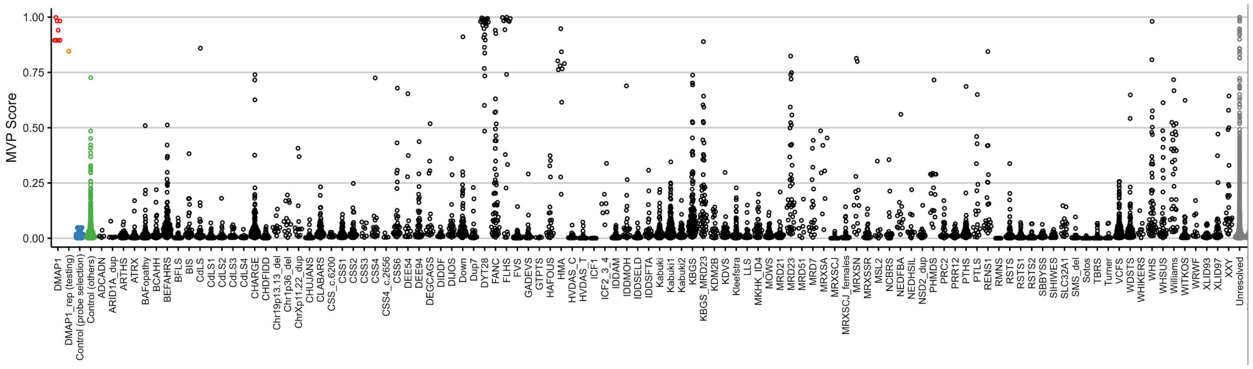

Supplemental Figure 7. Classification model trained using only matched controls. MVP scores generated by the SVM model, trained exclusively on *DMAP1* samples and their matched controls.
